# Gadolinium-based MR cisternography with prepontine cisternal routine for evaluating distribution pattern of intrathecal targeted drug delivery in pain management

**DOI:** 10.1080/10717544.2023.2189588

**Published:** 2023-03-17

**Authors:** Haocheng Zhou, Dingquan Zou, Bo Hong, Rong Hu, Tongbiao Yang, Xinning Li, Xin Li, Junjiao Hu, Ruixuan Wang, Yaping Wang

**Affiliations:** a Department of Pain, The Third Xiangya Hospital and Institute of Pain Medicine, Central South University, Changsha, China; b Hunan Key Laboratory of Brain Homeostasis, Central South University, Changsha, China; c Department of Pain Management and Anesthesiology, The Second Xiangya Hospital, Central South University, Changsha, China; d Department of Pain Management and Anesthesiology, Yueyang Traditional Chinese Medicine Hospital, Yueyang, China; e Department of Pain, Yongzhou Central Hospital, Yongzhou, China; f Department of Radiology, The Second Xiangya Hospital, Central South University, Changsha, China; g Bourns Engineering, The University of California, Riverside, Riverside, California, USA

**Keywords:** Gadolinium, cisternography, prepontine cisternal, distribution, intrathecal targeted drug delivery

## Abstract

Gadolinium-based MR cisternography has been mainly applied in clinical evaluation of cerebrospinal fluid leaking, that is conducted by intrathecal administration of contrast media. Recently, we have reported one novel technique of intrathecal targeted drug delivery with prepontine cisternal routine to treat orofacial cancer pain. The aim of this study was to examine the distribution pattern of this intrathecal drug delivery strategy. Here, we introduce one case who suffered severe orofacial pain caused by sublingual gland tumor, and successfully attenuated by prepontine cisternal administration of analgesic agents. To assess the distribution of intrathecal drugs, postoperative MR images of brain, cervical, thoracic, and lumbar segments in axial, coronal, and sagittal planes were obtained after application of gadolinium. The perfusion rate of contrast medium was set at 0.01 mmol per hour for 24 hours prior to MR scanning. In the T1-weighted images, we can identify contrast spread not only locating around the site of the intrathecal catheter tip, but also concentrated to the lateral sides. None obvious side effect was found after intrathecal injection of contrast media. Thus, our finding demonstrated the local distribution phenomenon of intrathecal drugs through prepontine cisternal access, and the bilateral perfusion pattern may provide insights underlying the analgesic mechanism of trigeminal pain provided by this novel intrathecal therapy. Gadolinium-based MR cisternography may serve as a potential tool to confirm the therapeutic effect of intrathecal targeted drug delivery via prepontine cisternal routine in orofacial pain management.

## Introduction

Gadolinium-enhanced MR cisternography is an imaging technique that applying intrathecal contrast material into the subarachnoid space, to evaluate the abnormality of the cerebrospinal system (Algin & Turkbey, 2013; Dogan et al., 2020). In addition to diagnostic value, intrathecal injection of contrast can provide accuracy and confirmation of epidural drugs in pain management, which has been commonly applied in lumbar level of spinal cord (Boies et al., 2022). Likely, a lumbar puncture procedure is considered to delivery contrast into the subarachnoid space before MR cisternography. However, the validation of cisternographic imaging in pain practice remains uncertain.

Recently, we have reported a novel technique of intrathecal targeted drug delivery (ITDD) via prepontine cisternal routine in orofacial cancer pain management (Zhou et al., 2022). By placing intrathecal catheter tip into the prepontine cisternal space, analgesic agents may directly target the cranial nerves, particularly approaching the trigeminal nerve system, which mainly governs the sensory input of orofacial region. Consequently, the intrathecal distribution pattern of drugs is a key factor for pain management (Prager et al., 2014). Thus, we assume that gadolinium-enhanced MR cisternography may be a feasible and effective approach to confirm the distribution pattern of medications in the prepontine cisternal space. In this study, we reported one case with severe orofacial pain caused by sublingual gland cancer, and achieved satisfactory control of pain after implantation of prepontine cisternal ITDD. To evaluate the distribution pattern of intrathecal medications, gadolinium-enhanced MR cisternography was performed postoperatively.

## Methods

### Case description

An eighty-two years old Chinese female who suffered severe orofacial pain caused by malignant tumor of sublingual gland, came to our pain clinic for pain control. The patient did not undertake any anti-tumor therapy before hospitalization. Severe background pain was reported by this patient with a mean visual analogue scale (VAS) of 7/10, and about five episodes of breakthrough cancer pain per day. Oral administration of oxycodone was applied 80 mg per day. The procedure was conducted in accordance with the Declaration of Helsinki, and ethics approval was not required for the retrospective design of study. Written consent was obtained by the patient prior to surgery and MR scanning.

### ITDD procedure

**Figure 1.**
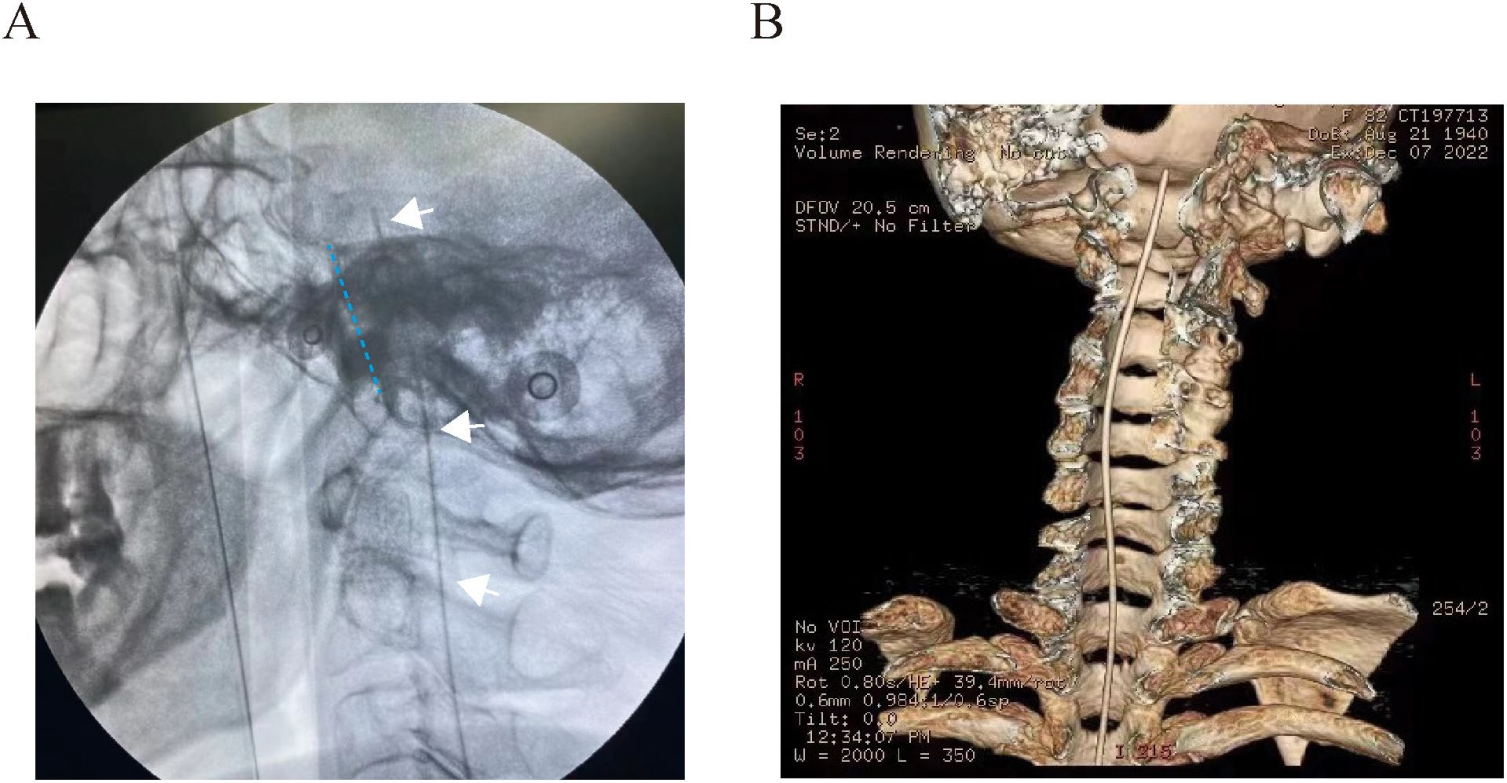
Intra- and post-operative confirmation of intrathecal catheter placement. (A) Depth of intra-prepontine cisternal catheter was positioned at the clivus level during procedure. (B) Postoperative three-dimensional reconstruction of intrathecal catheter. White arrow indicated the intrathecal catheter, and the blue dash line for the clivus respectively.

The surgical detail of ITDD has been described previously (Zhou et al., 2022). Specifically, patient was positioned in a left lateral decubitus position under general anesthesia. A lumbar puncture was performed at L2/3 level for cannulation of subarachnoid space under the guidance of fluoroscopic imaging. A 65-centimeter length intrathecal polyurethane catheter (ZS2, Linhwa, China) was then inserted into the subarachnoid space and advanced under lateral view of fluoroscopy. The target of intrathecal catheter tip was placed at clivus level (Figure 1a) according to our previous data (Zhou et al., 2022). To administrate intrathecal drug delivery, the distal ending of intrathecal tube was attached to an implantable port (ZS2, Linhwa, China), which was connected to one external electronical pump (ZZB-150, Aipeng, China) to control the release rate of medications. Patient was asked to stay in bed for at least two days to prevent headache caused by lumbar puncture.

### Postoperative computed tomography for three-dimensional re-constriction

To confirm the proper location of ITDD system, postoperative computed tomography was scheduled within one week after procedure. The imaging test was conducted in accordance with our previous protocol (Wang et al., 2021; Zhou et al., 2022). Helical images were obtained between the lumbar spinal cord and calva line. Imaging data were then transferred to the imaging workstation for three-dimensional reconstruction of ITDD (Figure 1b).

### Detail of intrathecal administration of drugs

A minimal dosage of intrathecal morphine (0.03 mg per day) was applied based on our previous experience (Zou et al., 2021; Zhou et al., 2022). Before accomplishment of morphine titration, patient kept oral administration of morphine and gradually reduced the dosage, and the intrathecal delivery rate of morphine was then adjusted according to the pain severity. For acquisition of cisternograpic MR imaging, gadopentetate dimeglumine has been considered as the safest and most recommended intrathecal contrast agent in human research (Albayram et al., 2008; Algin & Turkbey, 2013; Hagedorn et al., 2019). A total volume of 20 ml gadopentetate dimeglumine (Magnevist; Schering, Berlin, Germany) was diluted into 100 ml saline. The injection speed of intrathecal volume was set 0.1 ml per hour. Consequently, the infusion rate of contrast media was 0.01 mmol every hour approximately, and the total amount injected into the prepontine cisternal space was about 0.25 mmol.

### Gadolinium-enhanced MR cisternography

Intrathecal gadopentetate dimeglumine was administrated 24 hours prior to MRI scanning. Patient was positioned with one a supine position during imaging scanning, and orthogonal planes were scanned from lumbar, thoracic, cervical spinal cord to brain segment. Coronal, sagittal, and axial T1-weighted images were then obtained with a T1-MPRAGE-SAG-ISO sequence (2200/3.4, TR ms/TE ms), by using a 3.0 T MR unit (Vida, Siemens Healthcare, Erlangen, Germany).

## Results

### Therapeutic effect of ITDD

Pain symptom was satisfactorily attenuated by the intrathecal treatment, with reduction of pain scores over 50%, that mean VAS decreasing from 7 to 3 before discharge. In addition, the frequency of breakthrough was also reported significantly less than that before ITDD implantation. Two follow-ups were conducted through telephone interview at one-, and four-week after surgery, respectively. Only mild pain (VAS ranging between 1 to 2) was reported at each point, without obvious complain of breakthrough pain episodes. The intrathecal dosage of morphine was set 0.08 mg every 24 hours, and gradually increased to 0.15 mg one week after discharge, and 0.75 mg at one-month follow-up.

### Distribution pattern of gadopentetate dimeglumine

#### Coronal view of MR cisternography

**Figure 2.**
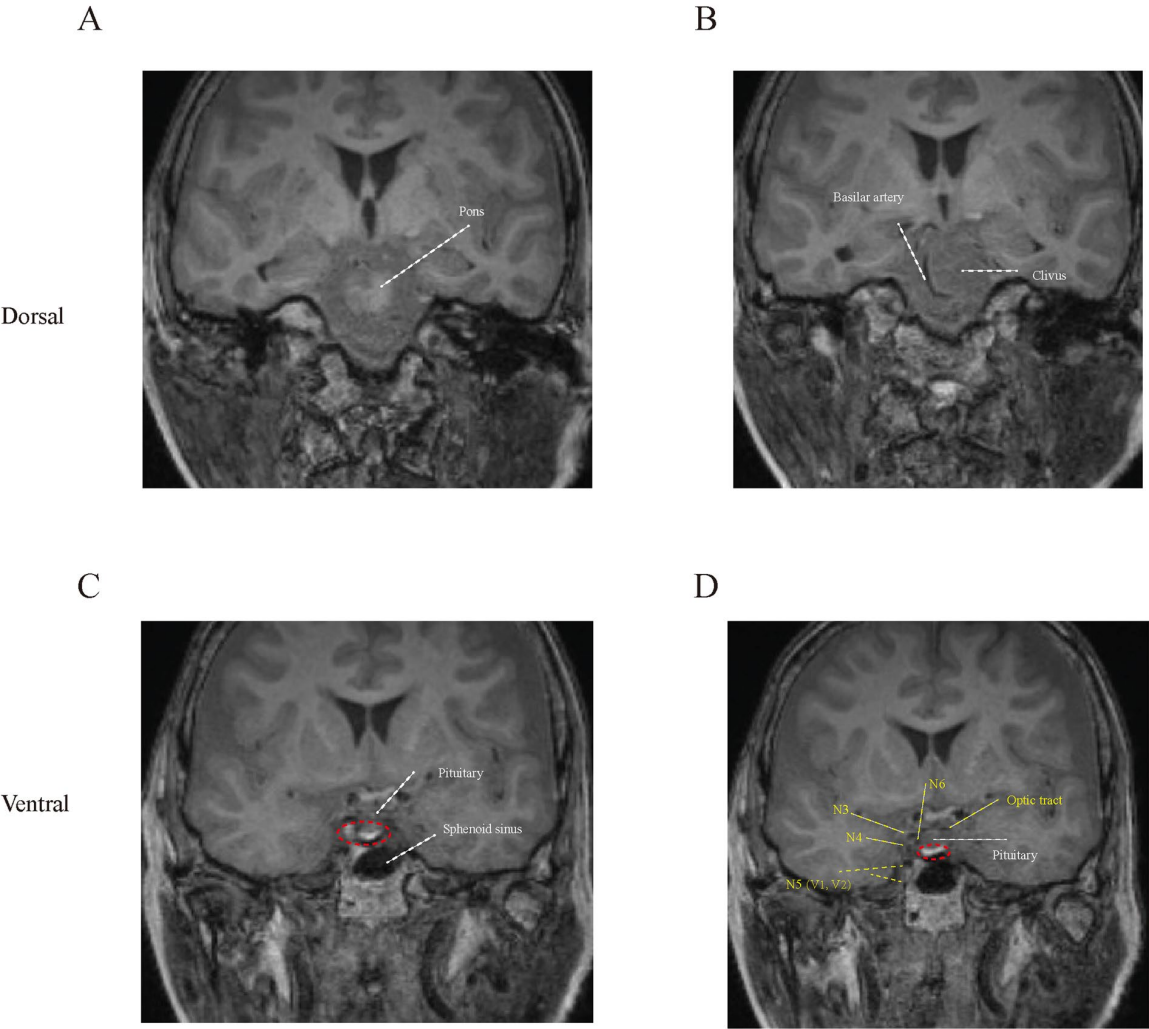
Coronal view of MR cisternography. (A–D) Dorsal to ventral sections of prepontine cisternal scanning. The red circle indicated the enhancement of gadolinium contrast.

Prepontine cisternal space is located dorsally to the clivus and ventrally to the pons, (Zhou et al., 2022) thus, we initially identified the position of pons and clivus at coronal section of MR cisternography (Figure 2a, b). At plane of pituitary, we can find enhancement of contrast in the prepontine cisternal cavity (Figure 2c), which subsequently covering the distribution region of cranial nerves, including oculomotor, trochlear, ophthalmic and maxillary branch of trigeminal, and abducent nerve (Figure 2d).

#### Gadolinium-enhanced horizontal imaging

**Figure 3.**
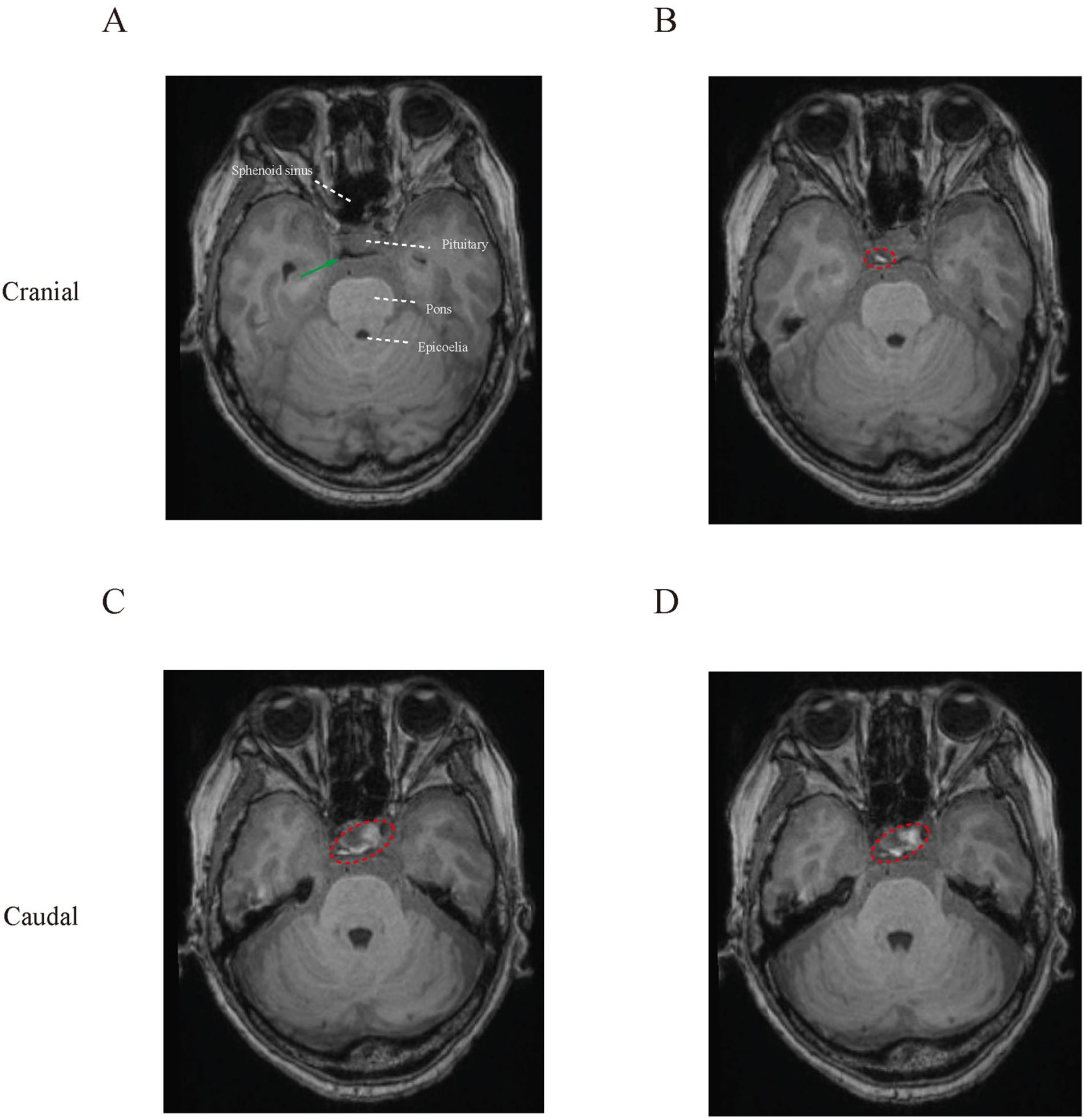
Horizontal spread of contrast media. (A) Top of prepontine cisternal space was initially identified at transverse plane, indicated by the green arrow. (B–D) Distribution of intrathecal agents at the anterior-posterior position.

To evaluate the horizontal pattern of contrast spread, we reviewed multiple transverse sections from the top of the prepontine cisternal cavity (Figure 3a). The upper part was filled with contrast unilaterally (Figure 3b), and gadolinium media spread bilaterally in the inferior prepontine cisternal portion (Figure 3c, d).

#### Sagittal spread of intrathecal drugs

**Figure 4.**
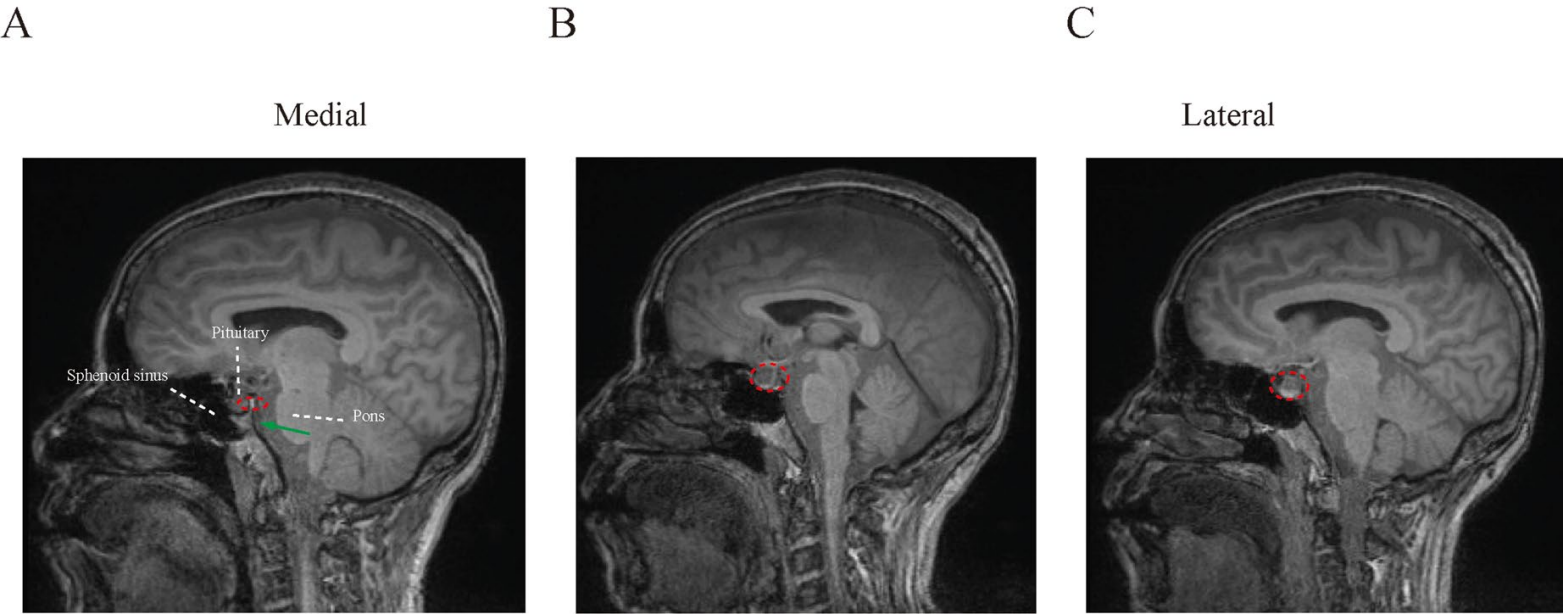
Sagittal spread pattern of intrathecal drugs. (A) Medial section of prepontine cisterna, and contrast spread ventrally in the lateral sections of subarachnoid cavity (B, C).

Next, we examined the distribution of intrathecal agents from medial plane of prepontine cistern (Figure 4a). At this section, the contrast was localized dorsally to the pituitary, as indicated by the circle in Figure 4(a). Contrast gradually covered the ventral pituitary in the lateral sections (Figure 4b, c).

#### Adverse effect of MR cisternography

We did not observe any obvious adverse reaction during intrathecal administration of gadolinium media (e.g. headache, nausea, vomiting, seizures, visual impairment, or mental changes) in this case.

## Discussion

Cisternograpic testing has been mostly used in diagnosis of cerebrospinal fluid leak in the last two decades. In general, a lumbar puncture procedure is considered for intrathecal delivery of contrast media (Albayram et al., 2008; Algin & Turkbey, 2013). To our knowledge, it is the for first time that we report a novel procedure access for gadolinium-based MR cisternography through prepontine cisternal routine. Our data demonstrated the feasibility of prepontine cisternal drug administration for intrathecal contrast-based MR imaging in human study.

Despite puncture access, the volume of contrast was significantly reduced in prepontine cisternal approach compared with the other spinal segment. For instance, the median volume of isovue administrated was 1.50 ml (ranging between 1–2 ml) to confirm the spread patterns of cervical epidural space (Gill et al., 2017). To perform gadolinium-based MR cisternography for detection of cerebrospinal abnormality, a single usage of 0.5 ml contrast was injected into the lumbar subarachnoid cavity respectively (Albayram et al., 2008). In contrast, the total amount of gadopentetate dimeglumine delivered into the prepontine cisternal space was about 0.25 mmol (volume of 0.25 ml), which is a safe and recommended dose in IDDS dye studies (Hagedorn et al., 2019). Meanwhile, the contrast we used was only about 2.5–15% of total amount of intrathecal gadolinium dimeglumine (Magnevist) reported in previous practices, to avoid potential neurotoxicity of contrast media (Hagedorn et al., 2019).

In addition to injection volume, the controlled release rate provided by ITDD system may also influence the spread pattern of contrast, compared with traditional single injection. Perfusion rate of gadolinium was set 0.01 mmol per hour, far less than the exact amount above. However, the protocol we used may not provide detail of cerebrospinal fluid disorder, due to the relatively small amount of contrast. Also, the elimination of drug driven by the vascular structures in the cerebrospinal system, (Mestre et al., 2018) may also hinder the diagnostic value of prepontine cisternal delivery. One obvious advantage of low dosage contrast is to reduce the potential risk of neurotoxicity (Hagedorn et al., 2019). In this case, we did not observe any obvious neurological complications of intrathecal gadolinium application.

Intrathecal parameters (e.g. volume, speed, analgesics dosage, and catheter location) are important factors for therapeutic effect. The initial intrathecal morphine started with a minimal dosage, and gradually increased with reduction of oral opioids based on our previous protocol (Zou et al., 2021). The transition between oral and intrathecal morphine usage was accomplished one month after surgery, with significant relief of resting and breakthrough cancer pain. In previous imaging examination, we found that the tip of catheter was located unilaterally in the prepontine cisternal space, which can be confirmed by the three-dimensional re-construction of computed tomography postoperatively (Zou et al., 2021). However, we think it unnecessary to insert the catheter tip into the painful site due to the circulation of cerebrospinal fluid. Furthermore, we can only judge the depth of catheter tip during surgery, but not implantable sites with lateral view of fluoroscopy. CT scanning demonstrated that the tip was closed to the medial line in this case. One main limitation of computed tomography is that the lack of sensitivity of brain structures and cerebrospinal fluid. Thus, we think it reasonable to apply MRI technique to investigate the distribution pattern of intrathecal drug delivery via prepontine cisternal access.

In the T1-weighted images, the spread of contrast in the subarachnoid space can by tracked. Enhanced signal was distributed bilaterally in the coronal (Figure 2c) and horizontal (Figure 3c, d) planes. This finding is consistent our hypothesis that unilateral placement of catheter tip can provide sufficient pain relief regardless of the site of painful lesion. Unlike ITDD, the therapeutic site and segment of spinal cord should be carefully designed for implantation of electrical nerve stimulation, to achieve optimal coverage of painful region (Zhou et al., 2022). Although the contrast media was transferred bilaterally, the area of enhancement was unequally distributed in the transverse section, that the ventral part of left subarachnoid space was more enhanced compared with the other hemisphere (Figure 3c, d). Further study may be needed to confirm the role of distribution in pain attenuation.

The fifth cranial nerve is located in the prepontine cistern, which receives predominate sensory input of the orofacial region. Thus, blockage of trigeminal nerve system may provide pain in patient with orofacial tumor. It is for the first time that our data provided graphic evidence of analgesic agents that directly targeted the branches of trigeminal branches (Figure 2c, d). In addition to trigeminal nerves, multiple cranial nerves may be covered by prepontine cisternal drug delivery, including abducens, oculomotor, and trochlear nerves. In general, major role of these cranial nerves serve as motor function, however, several cases reported involvement of oculomotor and trochlear nerves in neuropathic pain development (Nandana et al., 2021; Akhondian et al., 2022). Thus, we assume potential indications of this novel intrathecal therapy strategy for painful ophthalmoplegia with analgesic or local anesthetics.

Despite peripheral mechanism, (Stein, 1995) activation of opioid receptors at dorsal horn of spinal cord and brain tissue may also contribute to the analgesic function of ITDD treatment. Enriched expression of u-opioid receptor was determined across multiple brain regions, including cortex, striatum, hippocampus, locus coeruleus, and the superficial layer of spinal cord dorsal horn (Arvidsson et al., 1995). Delta and kappa ligands are active as well with relatively weaker affinity than mu-receptors, that functions analgesic effect in the central nerve system.Fowler and Fraser, 1994 Additionally, neural connection between trigeminal nerve system and high order of cervical spinal cord (C1-C4) may also serve potential target for intrathecal therapy in orofacial pain management (Taren & Kahn, 1962; Yalamuru et al., 2022). However, cervical involvement underlying pain relief provided by prepontine cisternal ITDD remains uncertain based on our current data, given the local distribution of intrathecal drugs.

## Conclusions

To our knowledge, it is for the first time that gadolinium-based MR cisternography was performed with prepontine cisternal drug delivery routine. It is feasible to apply this novel technique in pain practice to confirm the distribution pattern of intrathecal agents. Furthermore, our data revealed a dual-site but unequal model of drug distribution provided by ITDD system, which may underly the mechanism of analgesic effect for craniofacial pain. Further study with larger sample size is essentially needed to assess the pharmacokinetics of intra-prepontine drug application, distinct ITDD parameters like injection speed, volume, duration, and catheter tip location should be considered in the future.

## References

[CIT0001] Akhondian J, Ashrafzadeh F, Seilanian Toosi F, et al. (2022). Recurrent painful ophthalmoplegic neuropathy with unilateral oculomotor and trochlear nerve palsy in an 8-year-old girl. J Binocul Vis Ocul Motil 72:1–6. 35867412

[CIT0002] Albayram S, Kilic F, Ozer H, et al. (2008). Gadolinium-enhanced MR cisternography to evaluate dural leaks in intracranial hypotension syndrome. AJNR Am J Neuroradiol 29:116–21. 17947371 10.3174/ajnr.A0746 PMC8119121

[CIT0003] Algin O, Turkbey B. (2013). Intrathecal gadolinium-enhanced MR cisternography: a comprehensive review. AJNR Am J Neuroradiol 34:14–22. 22268089 10.3174/ajnr.A2899 PMC7966334

[CIT0004] Arvidsson U, Riedl M, Chakrabarti S, et al. (1995). Distribution and targeting of a mu-opioid receptor (MOR1) in brain and spinal cord. J. Neurosci 15:3328–41. 7751913 10.1523/JNEUROSCI.15-05-03328.1995 PMC6578209

[CIT0005] Boies B, Bhinder A, Probert S, Nagpal AS. (2022). Intrathecal contrast spread pattern in the lumbar spine in contralateral oblique view. Pain Med 23:1027–8. 34791433 10.1093/pm/pnab323

[CIT0006] Dogan SN, Salt V, Korkmazer B, et al. (2020). Intrathecal use of gadobutrol for gadolinium-enhanced MR cisternography in the evaluation of patients with otorhinorrhea. Neuroradiology 62:1381–7. 32535661 10.1007/s00234-020-02463-3

[CIT0007] Fowler CJ, Fraser GL. (1994). Mu-, delta-, kappa-opioid receptors and their subtypes. A critical review with emphasis on radioligand binding experiments. Neurochem Int 24:401–26. 7647696 10.1016/0197-0186(94)90089-2

[CIT0008] Gill J, Nagda J, Aner M, Simopoulos T. (2017). Cervical epidural contrast spread patterns in fluoroscopic antero-posterior, lateral, and contralateral oblique view: a three-dimensional analysis. Pain Med 18:1027–39. 28339542 10.1093/pm/pnw235

[CIT0009] Hagedorn JM, Bendel MA, Moeschler SM, et al. (2019). Intrathecal ­gadolinium use for the chronic pain physician. Neuromodulation 22:769–74. 31448498 10.1111/ner.13043

[CIT0010] Mestre H, Tithof J, Du T, et al. (2018). Flow of cerebrospinal fluid is driven by arterial pulsations and is reduced in hypertension. Nat Commun 9:4878. 30451853 10.1038/s41467-018-07318-3 PMC6242982

[CIT0011] Nandana J, Nair SS, Girdhar S, Sundaram S. (2021). Recurrent painful ophthalmoplegic neuropathy: a cause for recurrent third nerve palsy in a child. BMJ Case Rep 14:e246179. 10.1136/bcr-2021-246179 PMC8587473 34764123

[CIT0012] Prager J, Deer T, Levy R, et al. (2014). Best practices for intrathecal drug delivery for pain. Neuromodulation 17:354–72. 24446870 10.1111/ner.12146

[CIT0013] Stein C. (1995). The control of pain in peripheral tissue by opioids. N Engl J Med 332:1685–90. 7760870 10.1056/NEJM199506223322506

[CIT0014] Taren JA, Kahn EA. (1962). Anatomic pathways related to pain in face and neck. J Neurosurg 19:116–21. 13919670 10.3171/jns.1962.19.2.0116

[CIT0015] Wang Q, Chen C, Guo G, et al. (2021). A prospective study to examine the association of the foramen ovale size with intraluminal pressure of pear-shaped balloon in percutaneous balloon compression for trigeminal neuralgia. Pain Ther 10:1439–50. 34460076 10.1007/s40122-021-00311-7 PMC8586299

[CIT0016] Yalamuru B, Weisbein J, Pearson ACS, Kandil ES. (2022). Minimally-invasive pain management techniques in palliative care. Ann Palliat Med 11:947–57. 34412500 10.21037/apm-20-2386

[CIT0017] Zhou H, Han R, Chen L, et al. (2022). Effect of implantable electrical nerve stimulation on cortical dynamics in patients with herpes zoster-related pain: a prospective pilot study. Front Bioeng Biotechnol 10:862353. 35651542 10.3389/fbioe.2022.862353 PMC9149165

[CIT0018] Zhou H, Huang D, Zou D, et al. (2022). Prepontine cisternal routine for intrathecal targeted drug delivery in craniofacial cancer pain treatment: technical note. Drug Deliv 29:3213–7. 36261927 10.1080/10717544.2022.2134507 PMC9586698

[CIT0019] Zou D, Zhang W, Wang Y. (2021). Prepontine cistern intrathecal targeted drug delivery for cancer-related craniofacial pain. Pain Med 22:3112–4. 33620465 10.1093/pm/pnab059

